# F15. DIFFERENTIAL EXPRESSION PATTERNS OF EPIDERMAL GROWTH FACTOR (EGF) AND IMMUNE SYSTEM MARKERS IN DORSOLATERAL PREFRONTAL (BA46) AND ORBITOFRONTAL (BA11) CORTICES IN SCHIZOPHRENIA AND MOOD DISORDER

**DOI:** 10.1093/schbul/sby017.546

**Published:** 2018-04-01

**Authors:** Tharini Ketharanathan, Avril Pereira, Ian Everall, Suresh Sundram

**Affiliations:** 1 University of Melbourne; 2 Institute of Psychiatry, Psychology & Neuroscience, King’s College London; 3 Monash University

## Abstract

**Background:**

Environmental risk factors that operate at maternal, foetal and post-natal levels, causing immune activation are known risk factors for schizophrenia. How this risk is transduced is unknown but one plausible disease mechanism may be through immune activation perturbing central nervous system growth factor systems, such as the epidermal growth factor (EGF) system critical to neuronal differentiation, maturation and plasticity, altering neurodevelopment. These interactions between EGF and immune systems may involve specific critical brain regions and not others.

**Methods:**

The expression of candidate genes from EGF and immune systems and related signalling pathways, including ligands, receptors and intermediary molecules, were examined in post-mortem dorsolateral prefrontal (DLPFC) (n=114 genes) and orbitofrontal cortical (OFC) (n=105 genes) tissues, from schizophrenia and mood disorder patients and healthy controls (n=68), using the Fluidigm Biomark qRT-PCR platform. Data were analysed by ANOVA or corresponding non-parametric test (Kruskal-Wallis) in GraphPad Prism 6/7 statistical software and p values were adjusted for multiple testing (Benjamini-Hochberg).

**Results:**

In DLPFC, 68 genes were significantly differently expressed between diagnostic groups. In comparison to healthy controls, 60 genes were differentially expressed in schizophrenia and 14 in the mood disorder group. Collectively these differentially expressed genes belonged predominantly to ERBB signalling and associated MAPK, PI3K and MTOR pathways and with immune pathways involving toll like receptor (TLR), TNF, nuclear factor kappa B (NFB), JAK-STAT, and complement signalling. Although there was some overlap the expression profiles in schizophrenia and mood disorder differed considerably. There were genes (n=15) with significantly lowered expression in both patient groups compared to controls which belonged predominantly to immune pathways such as TNF and TLR. However, 36 genes were differentially expressed between schizophrenia and mood disorder, all of them having lower expression in schizophrenia, predominantly representing pathways PI3K/MTOR, MAPK, TLR and TNF signalling via NFKB. Gene expression in EGF system signalling via MAPK and PI3K pathways, and interleukin signalling via JAK-STAT were significantly lower in schizophrenia than in mood disorder and healthy controls. ErbB4 was the only gene significantly elevated in a patient group (mood disorder) compared to the controls.

In comparison to DLPFC, only five genes out of the 105 examined were differentially expressed between the diagnostic groups in OFC, which belonged to NFB, TLR, JAK-STAT and growth factor signalling pathways. In comparison to healthy controls all were differentially expressed in schizophrenia and three genes in the mood disorder group. The expression of most genes was decreased in the patient groups compared to control subjects in both brain regions.

**Discussion:**

We conclude that there is a prominent regional difference in the expression of EGF and immune system markers, identifying the DLPFC as a region of high activity for the interaction between these two systems relative to the OFC. In this region, the differing profiles of gene expression between schizophrenia and mood disorder involved EGF signalling pathways including PI3K/MTOR and MAPK along with immune pathways such as TNF, TLR and JAK-STAT signalling, possibly reflecting variant pathological processes involving immune and EGF system signalling between these sets of disorders.

